# Mapping endocrine networks by stable isotope tracing

**DOI:** 10.1016/j.coemr.2022.100381

**Published:** 2022-10

**Authors:** Ruth Andrew, Roland H. Stimson

**Affiliations:** University/ British Heart Foundation Centre for Cardiovascular Science, Queen's Medical Research Institute, University of Edinburgh, 47, Little France Crescent, Edinburgh, EH16 4TJ, United Kingdom

**Keywords:** Steroid, Stable-isotope, Flux, Mass spectrometry, Magnetic resonance, Vibrational spectroscopy, Positron emission tomography

## Abstract

Hormones regulate metabolic homeostasis through interlinked dynamic networks of proteins and small molecular weight metabolites, and state-of-the-art chemical technologies have been developed to decipher these complex pathways. Stable-isotope tracers have largely replaced radiotracers to measure flux in humans, building on advances in nuclear magnetic resonance spectroscopy and mass spectrometry. These technologies are now being applied to localise molecules within tissues. Radiotracers are still highly valuable both preclinically and in 3D imaging by positron emission tomography. The coming of age of vibrational spectroscopy in conjunction with stable-isotope tracing offers detailed cellular insights to map complex biological processes. Together with computational modelling, these approaches are poised to coalesce into multi-modal platforms to provide hitherto inaccessible dynamic and spatial insights into endocrine signalling.

## Highlights

• Stable isotope tracing is an exceptionally valuable tool to study metabolic flux *in vivo* in humans.
• Fluxomics can identify and quantify integrated endocrine pathways via tracking labels through multiple metabolic networks.
• Improved sensitivity and resolution of nuclear magnetic resonance spectroscopy and mass spectrometry, have enhanced the detection of labelled tracers.
• Spatial mapping of stable-isotope tracers in tissues and living cells can be achieved by mass spectrometry imaging and vibrational spectroscopy.
• Radiotracers combined with 3D imaging, such as positron emission tomography, offer high resolution with *in vivo* tracing.

Current knowledge of biochemical pathways underpinning the hormonal control of metabolism stems from detailed dissection of chemical intermediates *in vitro*, *ex vivo* and *in vivo* by pioneers such as Randle et al. [1] and Warburg [2]. Early research involved careful chemical elucidation of species derived from blood and bioactive extracts of endocrine organs, for example adrenal extracts studied by Osler [3]. Chromatography, then in paper and thin layer modalities, enabled identification against standard compounds which were often synthesised bespoke with stereochemical precision in-house. These pivotal experiments and many others, painstakingly executed, have laid the bedrock of our current understanding of flux in metabolic endocrinology. In the last century, technological and computational advances have transformed our abilities to study multiple dynamic pathways concomitantly using isotopically labelled tracers, consolidating fields such as metabolomics and fluxomics [4]. In this article, we review the recent advances (focussing on publications since 2019) in the study of metabolic flux in endocrine science and highlight the current challenges and future opportunities (Table 1).

**Table 1:** Comparison of advantages and disadvantages of techniques used in fluxomics and *in vivo* tracing.

Technology	Stable or radio-isotope	*In* or *ex vivo*	Advantages	Disadvantages	Citations
NMR Fluxomics	Stable	*Ex vivo*	- Minimal sample processing - Non-destructive - Fluids and tissue possible - Rapid sample analysis	- Lower sensitivity than MS - Narrow coverage of metabolome - Limited stable-isotope tracers detectable - Costly tracers	[37, 38, 39, 40, 41]
MS Fluxomics	Stable in man Radio in animals	*Ex vivo*	- More sensitive than NMR - Broader coverage of metabolome than NMR - More complex sample preparation than NMR - Cellular spatial resolution with imaging - Wider range of stable isotope tracers than NMR	- Slower than NMR - Destructive - Limited stable-isotope tracers - Costly tracers - Quality control of batches challenging	[34∗∗, 35, 36]
PET Tracing	Radio	*In vivo*	- High sensitivity - Quantifies tissue tracer uptake - Anatomical localisation of whole body tissue uptake through combination with CT/MRI - Can map receptors/transporters through use of specific tracers	- Low spatial resolution - Radiation exposure - Challenges synthesising tracers - Not all molecules amenable to labelling - Cannot distinguish tracer and metabolites - Requires cyclotron close by - Limited number of concomitant tracers - Expensive	[19∗, 20, 21, 22, 23]
MRI/MRS Tracing	Stable	*In vivo*	- Non-invasive - Organ specific - Quantitation of multiple metabolites - Can assess dynamic changes in metabolism	- Low sensitivity - Band broadening caused by matrix - Limited number of isotopes amenable to tracing, requiring specific coils	[10,11,24, 25, 26, 27, 28, 29, 30, 31, 32, 33]
Raman Imaging/Spectroscopy	Stable	Cells	- Selective spectra of tracers - Multiple vibrational probes measured together - Sub-cellular spatial resolution - Non-destructive - Measurement in multiple matrices - Rapid	- Low sensitivity - Requires extensive instrumental tuning to maximise sensitivity	[65, 66, 67, 68, 69, 70, 71, 72, 73, 74, 75, 76, 77, 78, 79, 80, 81∗∗, 82, 83, 84]

Abbreviations: CT= computerised tomography; MRI = magnetic resonance imaging spectroscopy; MS = mass spectrometry, NMR: nuclear magnetic resonance, PET, positron emission tomography.

## Navigating the endocrine web of knowledge

Biochemical pathways are networks of multiple interlinked steps, catalysed by many enzymes, each with their own kinetic characteristics. Diseases of ‘inborn errors of metabolism’ exemplify this complex interplay, where phenotypes of congenital adrenal hyperplasia for example differ depending on which adrenal enzyme is mutated [5]. To link pathophysiological features with biochemical kinetics, families of closely related molecules can be quantified, creating time-stamped fingerprints of health and disease. While quantifying absolute levels of biochemical species at single timepoints is challenging in its own right, these measurements mask the natural turbulence in flow through the overall labyrinth. A typical scenario is glucose homeostasis, where circulating glucose concentrations are very finely tuned, despite large changes taking place in flux through the pool. The rates of gluconeogenesis and glucose disposal constantly adjust to accommodate the nutritional challenges of daily life (e.g. feeding, exercise) and flux through the body pools must be dissected to understand metabolic health and disease.

**Figure 1:**
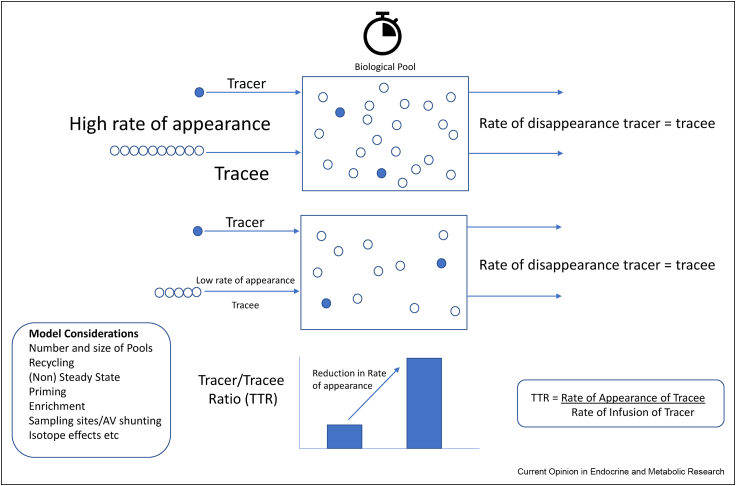
**Principles of isotope dilution at steady state.** To quantify the rate of appearance of an endogenous molecule, known as the tracee, an isotopically labelled tracer is administered at a fixed rate. The tracer is a version of the tracee which is labelled with a radio or stable isotope. Samples of the biological pool are obtained, and the proportion of tracer to tracee is quantified. This value can be used to calculate the rate of appearance of the tracer; taking into account, their rates of clearance are equivalent. The Tracer:Tracee ratio is used to calculate the rate of appearance of the tracee based on a known infusion rate of tracer. The equation given requires the model to meet a number of assumptions and more complex models have been derived accounting for a series of variable parameters, for example numbers and sizes of pools, tracer recycling, measurement in steady state or non-steady state conditions, the degree of enrichment and priming, whole body or tissue specific sampling by arterio-venous sampling and the effect of the presence of an isotope on the fate of the labelled versus the unlabelled species — this list is not exhaustive and the reader is directed to [12] for further details. Tracer blue circles, Tracee open circles. AV= arterio-venous.

Flux quantifies the rate of transit of a molecule through a biological pool depending on its rates of influx and efflux and can be assessed using tracers. The concept of tracing involves the administration of a proportionately small amount of a labelled version (tracer) of the endogenous molecules of interest at a known rate (Figure 1) and has been established for a century [6,7]. By knowing the rate of appearance of the tracer, the rate of appearance and disappearance of the endogenous species, termed the ‘tracee’, can be ascertained, either under steady or non-steady state conditions. Over the years, various tracers have been used in metabolic endocrinology, for example to determine the turnover of glucose [8,9], lactate and propionate [10] and glucocorticoids [11]. The mathematical principles underpinning these models are highly developed for abundant species such as glucose, glycerol, lactate, fatty acids and proteins and continue to be refined [10] and have been applied widely to answer numerous research questions, particularly related to metabolic disease, nutrition and exercise (for detailed theoretical principles see Wolfe and Chinkes [12], briefly summarised in Figure 1).

## Some like it hot

Early studies in the field relied on radioactive tracers, allowing radiolabelled atoms, usually tritium and ^14^carbon, to be tracked, either through retention in metabolites or loss to the environment through exhaled labelled CO_2_ and water. Endocrine examples include tracing steroid metabolism, for example oestrogens [13], androgens [14] and glucocorticoids [15], both *in vivo* and *ex vivo*. Today, the use of long-lasting radioisotopes is usually avoided in humans for ethical and safety reasons, although radioisotopes continue to offer attractive solutions for tracing *in vitro* and in animals. The strengths of the radiometric approach include the high specificity with which the tracer isotope can be identified against a non-radioactive, endogenous backdrop and also the high sensitivity of detection when coupled with extended counting times. Indeed, the measurements of radioactive glucose still underpin many recent studies of glucose flux and deoxyglucose disposal in mice [16], where sample size is limited. Furthermore, it is possible to discriminate co-administered tracers with two different radio-isotopic labels, for example tritiated glucose and 3-O-[^14^C]-methyl-d-glucose, which together can measure extracellular glucose kinetics as well as transport since they are not metabolised through the same pathways [17]. In current times, for safety reasons, these approaches with multiple radiotracers are more commonly applied in preclinical models [18]. However, for accurate quantitation, integrated chemical separation of the labelled precursor from its metabolites is necessary, a problem that still confounds the autoradiographic assessment of drug distribution.

**Figure 2:**
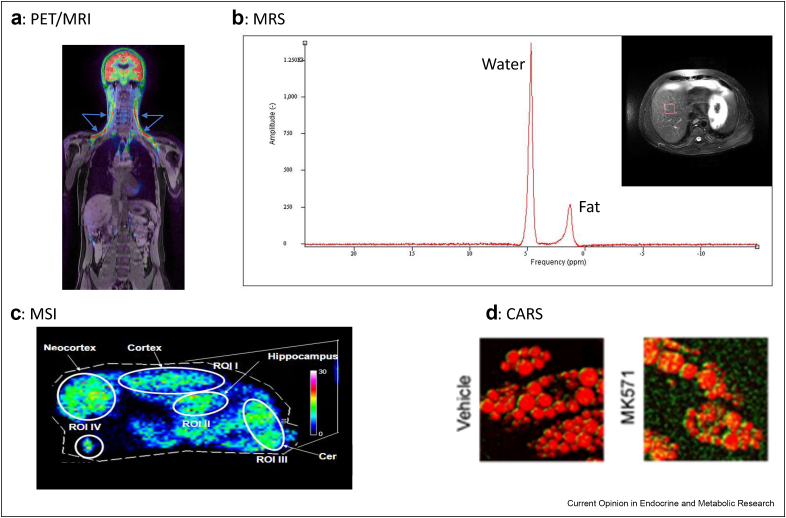
**Imaging modalities allowing isotope tracing.** (**a**) Fused ^18^F-fluorodeoxyglucose (^18^F-FDG) PET/MR image demonstrating significant ^18^F-FDG uptake by brown adipose tissue bilaterally in the cervical and supraclavicular regions (arrows). ^18^F-FDG PET exploits the high rates of glucose uptake and glycolysis by human brown adipose tissue during cold activation. Following transport into tissues, ^18^F-FDG is converted to ^18^F-FDG-6-phosphate which cannot readily continue through the glycolytic pathway, thus is ‘trapped’ and accumulates in the cell, making this a very sensitive technique to quantify glucose uptake. The image was obtained from a subject housed at 16 °C for 3 h, following 2 h of cold exposure 75MBq of ^18^F-FDG was injected intravenously with this image taken an hour later. (**b**) Magnetic resonance image and ^1^H-MR Spectroscopy spectra from a selected region of interest in the liver (square) obtained from a patient with type 2 diabetes mellitus, revealing high liver fat content. The water (∼4.7 ppm) and fat (∼2.3 ppm) peaks are shown on the spectra. T1 weighted images were obtained on a 1.5T GE MR scanner, spectra were obtained in a 30 × 30 × 30mm single voxel placed away from the major vessels using a PRESS sequence using a repetition time (TR) and echo time (TE) of 5000 and 40 ms, respectively. Thanks to Professor Mark Strachan and Dr Calum Gray, University of Edinburgh. (**c**) MS image collated using matrix-assisted laser desorption ionisation showing heatmaps representing [9,12,12-^2^H_3_]-cortisol regenerated in regions of interest (ROI) of a mouse brain (male), including cortex, hippocampus and cerebellum (Cer), following steady state infusion of [9,11,12,12-^2^H_4_]-cortisol and measured following derivatisation with Girard T. Full study reported in [59], but briefly, [9,11,12,12-^2^H_4_]-cortisol is infused as a tracer to measure regeneration of cortisol by the enzyme 11β-hydroxysteroid dehydrogenase type 1. [9,11,12,12-^2^H_4_]-cortisol is converted to [9,12,12-^2^H_3_]-cortisone by the enzyme 11β-hydroxysteroid dehydrogenase type 2, and [9,12,12-^2^H_3_]-cortisone is subsequently regenerated into active [9,12,12-^2^H_3_]-cortisol by the type 1 isozyme. Thus cortisol generated *de novo* by the adrenal gland can be distinguished from that generated in other tissues (e.g. brain, liver, adipose) by 11β-hydroxysteroid dehydrogenase type 1. This tracer has been valuable in understanding the role of this enzyme in diseases of metabolic or cognitive dysfunction and in characterising it as a potential drug target. (**d**) Coherent anti-Stokes Raman spectroscopy showing the images of adipocytes treated with [2,2,4,6,6,7,21,21-^2^H]_8_-(D_8_)-corticosterone with or without MK571, an inhibitor of the steroid transporter ABCC1. Resonance of the carbon-hydrogen bonds abundant in lipid is represented in red; carbon deuterium resonance from D_8_-corticosterone is represented in green and is more abundant with inhibition of export, notably in a distribution around the intracellular lipid droplets (scale bar, 20 μm). Reproduced under license number 5254281503812 [70]. Briefly, this approach has added value in understanding whether glucocorticoids pass into and out of cells by active and or passive transport. The historical view of the endocrine field has been that steroids passively traverse lipid membranes when travelling in and out of cells, but these recent studies suggest that ABCB1 and ABCC1 pumps have roles in actively exporting different glucocorticoids from cells where they are expressed.

Short-lived radioactive tracers have found a renaissance in conjunction with clinical and preclinical positron emission tomography (PET). PET is combined with either computer tomography or magnetic resonance imaging to localise tracer uptake and has distinct advantages as a non-invasive technique with high sensitivity and quantitative accuracy. The most commonly used PET tracer is [^18^F]-2-deoxy-2-d-glucose (^18^F-FDG) which is taken up and trapped by metabolically active tissues such as cancers. In addition, ^18^F-FDG-PET is now used to quantify glucose uptake by multiple metabolic tissues in both preclinical and clinical research [19] and has become the most commonly used technique to quantify activity in human brown adipose tissue, such as assessing regulation of this tissue by hormones such as glucocorticoids, and secretin [20,21](Figure 2a). Alternative PET radiotracers are also used clinically for certain endocrine conditions, such as ^18^F-fluorocholine which exploits the high phosphatidylcholine turnover in hyperparathyroidism [22], and gallium-68-DOTATE which binds to somatostatin receptors and is used to localise neuroendocrine tumours [23]. With imaginative chemistry, these and other radiotracers will be invaluable to further dissect endocrine pathways in health and disease *in vivo*. The field is only limited by the amenability of the molecules to be labelled by an appropriate short-lived isotope and the ability to rapidly synthesise the tracers in a time window compatible with the half-life. Research PET facilities, directed by specialist chemists and equipped with cyclotrons, are now more readily accessible.

## Stabilising the field

For studies of flux in humans, researchers have almost exclusively switched to using stable-isotope labelled tracers, which are becoming commercially available in increasing variety, although sometimes at substantial cost. These offer a safer alternative to radioactive labelling, although short-term side-effects can still arise, for example dizziness, when using heavy water. These products are also subject to strict guidance governing preparations for human use. Isotopes such as ^2^H and ^13^C are most common, but labelling of many other atoms, such as ^15^N or ^17/18^O, is possible with the quantification of dilution of these stable isotopes into the endogenous pool (Figure 1). Stable-isotopically labelled tracers continue to be employed extensively and innovatively to model specific pathways of metabolism, such as glucose in breath tests to detect early insulin resistance [24,25], lipids to assess remission of Type 2 diabetes [26], lactate in measuring liver metabolism [10] and glucocorticoids to assess potential new therapies for Type 2 diabetes [11,27], building on the extensive legacy of radiotracers. They have the advantage of revealing greater chemical detail. By labelling molecules such as glucose and fatty acids [28,29] with distinct label types and in different positions, flux through individual arms of biochemical pathways can be ascertained, often using fragmentation analysis. Indeed, differences in the rates of appearance of an endogenous molecule measured by tracers labelled in specific positions can reveal recycling through different biochemical shunts, for example fructose-6-phosphate with pioneering early studies in the endocrine field by Schulman et al. [30] but more recent examples across biology demonstrate its broader potential [31, 32, 33]. In contrast, radioactive tracers with distinct radioisotopes can be distinguished but the positions of the labels cannot be discerned easily.

## Scaling up the network

Fluxomics [4] is an extension of tracer profiling offering greater breadth of coverage. Instead of focussing on distinct biochemical families, this approach, benefitting from advances in the field of metabolomics, integrates flux through multiple pathways simultaneously and provides a bird's-eye view of the wider network. It is, however, important to note that species must display sufficient chemical similarity to be separated and detected together by the same analytical system. Thus a metabolomic profile is a bespoke subset of metabolites fitted to purpose, unlike the comprehensive and generic coverage gained by, for example whole genome sequencing. The values of flux obtained are semi-quantitative, with key findings requiring quantitative validation. Integrating stable isotopically labelled species into the metabolome allows the fate of biomolecules to be tracked by measuring the relative enrichment of metabolites with defined numbers of tracer atoms, and yielding information not always revealed through gene networks [34∗∗, 35, 36]. For example, the citric acid cycle involves disassembling C6 units and reassembling C3 subunits, the fate of which can be tracked to quite remote metabolites.

## Instrumental changes

Evident from its name, stable-isotope tracing should involve administering ‘trace’ amounts of tracer in comparison to the tracee into the biological pool of interest to avoid disturbing the natural kinetic rates. This has been made possible through advances in analytical technology allowing sensitive detection of tracers and distinction from non-labelled molecules and is largely reliant on mass spectrometry (MS) and magnetic resonance. Nuclear magnetic resonance (NMR) spectroscopy was one of the first approaches used in the metabolomics field, distinguishing stable isotopes with spin capability (such as ^1^H, ^13^C, ^15^N, ^19^F and ^31^P) from naturally abundant atoms and revealing their chemical environment within metabolites through specific chemical shift patterns. The approach is limited in sensitivity; albeit technology such as magnetic field strength continues to advance. To date in the endocrine field, it has largely been used to track more abundant species such as glucose, fructose, glycine, glutamate and glutamine [37, 38, 39, 40, 41] *ex vivo.* Fate mapping is possible, exemplified by the use of novel tracers, such as [2,3–^13^C]_2_-glucose, to distinguish the pentose phosphate pathway from glycolysis [42]. More recently, the rates of assembly of larger biomolecules such as RNA are being reported as amenable to tracing by NMR [43]. The extrapolation of NMR technology to magnetic resonance spectroscopy (MRS) offers exciting spatial opportunities *in vivo* (Figure 2b), although increased band broadening may result in over-lapping spectra [44], but further advances with higher field strengths continue to aid with discrimination. MRS, for example, has been useful to detect fluorinated drugs, including steroids [44,45]. This approach can be adapted to detect stable-isotope labelled tracers, although large amounts of tracers are required for detection. There are examples whereby mitochondrial oxidation has been visualised by MRS, using [1–^13^C]-acetate as a substrate, highlighting opportunities, but this field has not advanced substantially, being currently somewhat restricted by spatial resolution and cost [46, 47, 48]. The endocrine field is still to fully realise the opportunities of hyperpolarised carbon, which can boost signals by 4 orders of magnitude, albeit transiently [49] but examples are beginning to emerge in the endocrine cancer arena [50,51] and in the identification of defective cardiac metabolism in diabetic subjects [52]. The use of deuterated substrates by MRS to map dynamic metabolism through quantifying depletion of peaks of paired non-labelled metabolites within proton spectra also offer exciting future opportunities [53,54] for endocrine researchers.

**Figure 3:**
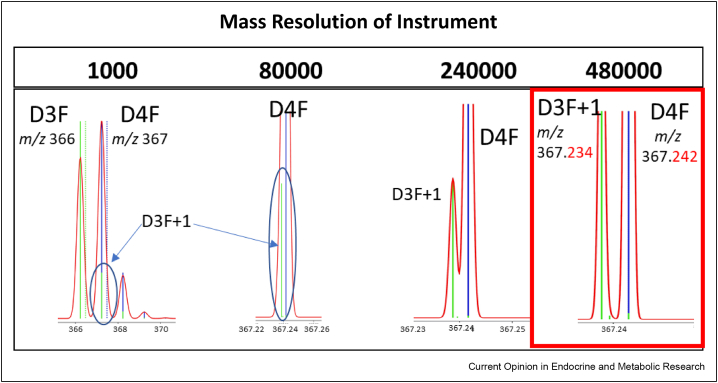
**The value of increasing the resolution of mass spectrometers on isotope distinction.** The panels show the added value of increasing mass resolution on the ability to distinguish isotopologues of [9,12,12-^2^H_3_] (D3F) and [9,11,12,12-^2^H_4_]-cortisol (D4F) used in the studies of appearance of cortisol [11]. The simulation of mass resolution (Mr) settings of 1000 (typical of quadrupoles instruments), 80000 (typical of time of flight (ToF) instruments and 240000 and 480000 (achievable by Orbitrap® and Fourier transform ion cyclotron resonance instruments) were performed using https://www.protpi.ch/. Nominal masses of protonated ions of D3F and D4F are *m/z* 366 and *m/z* 367 and that of natural ^13^C-D3F, also 367. Using quadrupole instruments (Mr 1000), D3F containing one natural 13-carbon (*m/z* 367.234; green) cannot be resolved from D4F (*m/z* 367.242; blue). ToF instruments with Mr 80000 are also unable to resolve the two species. However, resolution can be achieved partially with Mr 240000 and fully baseline resolved at 480000, achievable using Orbitrap and Fourier transform ion cyclotron resonance instruments. Thus, the use of high resolution instruments can reduce background noise and remove the requirement for mathematical correction.

MS approaches are more sensitive than NMR and readback wider profiles of metabolites but, when used ‘shotgun’ without further fragmentation or orthogonal separation techniques, cannot readily assign molecular stereochemistry, which is fundamental to intrinsic bioactivity. MS instruments have improved in their ion transfer capacities, ionisation efficiencies and detector sensitivity, all enabling detection of smaller amounts and thus lower tracer enrichments. Background noise has not only been reduced through improved extraction technologies to attenuate ion suppression but also by increasing engagement with high resolution instruments in quantitative analysis of small molecules. Quadrupole systems were the mainstay of tracer studies prior to metabolomic approaches [11] but could not distinguish deuterium from ^13^C by mass, meaning generalised correction factors had to be applied to account of natural isotopologues where mass differences were 2 or less. High resolution instruments have greater ability to distinguish isotopes, including deuterium and ^13^C (Figure 3), and are now available for both gas and liquid chromatographic applications, coupled with detectors suited for quantitation over the dynamic range matched to biological fluctuation. Tracking of metabolism of branched chain amino acids is an example of metabolic mapping by MS [55]. Fractional synthesis rates of larger molecules can also be measured, for example insulin [56]. Ideally tracing the metabolic fate of multiple isotopologues concomitantly would streamline analysis, reduce sample volumes and reduce batch effects, but this represents challenges in terms of duty cycle especially when using quadrupoles. The use of high mass resolution instruments can circumvent this problem somewhat but yield datasets of high complexity. Further advances, such as ion chromatography interfaces, are being explored in this technological space [57]. Notably, there are opportunities to combine MS and MRS for additional insight [58].

## Tracing in multi-dimensions

The MS approaches described thus far allow analysis *ex vivo* of fluids or tissue biopsies but lack spatial information. The emerging approach of MS imaging has the capacity to explore isotopic enrichment when coupled with high resolution detectors. The best known approach is matrix-associated laser desorption ionisation-MS imaging, which, for example, was used to trace stable-isotope labelled cortisol [59] within brain subregions (Figure 2c). It can also be applied in proteomic analysis of endocrine tissues [60] and exciting studies are emerging combining proteomics with isotope dilution analysis with a spatial dimension [61]. The spatial resolution of matrix-associated laser desorption ionisation-MS imaging is gradually improving, with sampling areas now reaching a <10 μm grid, so achieving cellular sampling voxels. This reduction in sampling area has to be balanced against diminishing amounts of analyte available for detection, but for highly abundant biomolecules, for example lipids, this technique can offer exquisitely detailed spatial maps which can then be integrated with transcriptomics profiles [62] and in the future co-registered with other histological sampling techniques such as spatial transcriptomics. Other ionisation sources, such as secondary ion mass spectrometry, also provide subcellular imaging of abundant molecules [63] and these opportunities are yet to be exploited to their full potential to assess flux in endocrine science. Researchers are also finding innovative solutions to assess stereochemistry, for example through the use of ozone to resolve lipid isomers [64].

## Good vibrations

Alternative chemical technologies hold untapped potential to detect tracers in a regional manner, for examples vibrational spectroscopy approaches, including Raman spectroscopy. These technologies distinguish the frequencies of vibrations of specific bonds which differ for heavy and natural isotopes and are thus amenable for stable-isotope probing and to study isotope exchange. Raman spectroscopy offers spatial information and importantly is non-destructive. Sensitivity is less than that of MS but advances, such as surface-enhanced Raman spectroscopy and Coherent anti-Stokes Raman continue to expand possibilities through improved signal to noise. Raman has been used most commonly to study cells and microbes, for example energy consumption [65] and spatial flux [66] pertaining to lipids [67,68] and glucose [69]. Reports using Raman to detect sterols and steroids are emerging [70] (Figure 2d). Cholesterol [71] with high abundance was initially most tractable. However, recent examples of detection in biomatrices include oestrogens [72], where oestradiol, oestrone and oestriol were distinguished [73]. Other examples include cortisol [74, 75, 76], progestogens [77] and androgens [78]. Beyond steroids, insulin secretion has been tracked using Raman spectroscopy [79] with broader applications appearing, for example in oocyte biology [80]. Raman has the capacity to concomitantly measure multiple vibrational probes exemplified by the use of stimulated Raman scattering [81, 82]. There are as yet limited examples of using isotopically tracers of steroids [70] and the extension of this technology *in vivo* is challenging [83], but this a field to watch [84].

## A dynamic future and resolutions for the coming years

In conclusion, the capacity to mine the biochemical flux through endocrine pathways has moved forward in leaps and bounds, supported by technological advances in applied analytical chemistry, but still based on the solid foundations laid in the field over the last century. Furthermore, exciting new developments in chemical detection continue to open new doors for spatial and real-time assessment across the full spectrum of experimental model systems from cells to humans. While flux analysis started with single families of molecules, the datasets now being generated through fluxomic approaches offer deeper dives into the metabolome but bring with them challenges in data analysis and standardisation. New software approaches to annotation and community standards are helping align the field [85,86], and the integration of NMR and MS approaches are proving valuable to fully mine datasets with simultaneous tracers [87]. With support from artificial intelligence and machine learning [88], spatial and dynamic multi-modal imaging are exciting fields we can eagerly anticipate in the coming decade.

## Funding

This research did not receive any specific grant from funding agencies in the public, commercial or not-for-profit sectors. RHS is funded by a senior fellowship from the Chief Scientist Office (SCAF/17/02).

## Conflict of interest statement

Nothing declared.
